# Thermogenesis in Adipose Tissue Activated by Thyroid Hormone

**DOI:** 10.3390/ijms21083020

**Published:** 2020-04-24

**Authors:** Winifred W. Yau, Paul M. Yen

**Affiliations:** 1 Laboratory of Hormonal Regulation, Cardiovascular and Metabolic Disorders Program, Duke NUS Medical School, Singapore 169857, Singapore; winifred.yau@duke-nus.edu.sg; 2 Duke Molecular Physiology Institute, Duke University, Durham, NC 27708, USA

**Keywords:** thermogenesis, thyroid hormone, brown adipose tissue, browning

## Abstract

Thermogenesis is the production of heat that occurs in all warm-blooded animals. During cold exposure, there is obligatory thermogenesis derived from body metabolism as well as adaptive thermogenesis through shivering and non-shivering mechanisms. The latter mainly occurs in brown adipose tissue (BAT) and muscle; however, white adipose tissue (WAT) also can undergo browning via adrenergic stimulation to acquire thermogenic potential. Thyroid hormone (TH) also exerts profound effects on thermoregulation, as decreased body temperature and increased body temperature occur during hypothyroidism and hyperthyroidism, respectively. We have termed the TH-mediated thermogenesis under thermoneutral conditions “activated” thermogenesis. TH acts on the brown and/or white adipose tissues to induce uncoupled respiration through the induction of the uncoupling protein (Ucp1) to generate heat. TH acts centrally to activate the BAT and browning through the sympathetic nervous system. However, recent studies also show that TH acts peripherally on the BAT to directly stimulate Ucp1 expression and thermogenesis through an autophagy-dependent mechanism. Additionally, THs can exert Ucp1-independent effects on thermogenesis, most likely through activation of exothermic metabolic pathways. This review summarizes thermogenic effects of THs on adipose tissues.

## 1. Introduction

### 1.1. Thermogenesis and Adipose Tissue

Thermogenesis is an essential survival mechanism for homeotherms. Obligatory thermogenesis is sufficient to maintain body temperature and normal body function when the ambient temperature is at thermoneutrality, which is about 23 °C for an adult man [1]. When ambient temperature falls below thermoneutrality, adaptive (also known as facultative) thermogenic mechanisms that require or do not require shivering are recruited. Shivering thermogenesis increases heat production in response to cold through skeletal muscle contraction. It is able to provoke a 5-fold increase in the resting metabolic rate in humans, and is important for surviving extreme cold conditions [2]. However, it lacks long-term sustainability and may compromise muscle function [3,4]. Non-shivering thermogenesis occurs mainly in brown adipose tissue (BAT) which is able to oxidize lipids and dissipate energy in the form of heat and is important for heat production during sustained cold exposure [5,6]. Shivering may be absent or become minimal when non-shivering thermogenesis is sufficient to generate heat, such as during moderate cold exposure or cold acclimation [7]. In homeothermic species, these processes are critical for maintaining normal body function and temperature.

There are two major types of adipose tissue: white adipose tissue (WAT) and brown adipose tissue (BAT), which stores energy and generates body heat, respectively. WAT is stored subcutaneously and viscerally, where it surrounds intra-abdominal organs such as the liver, pancreas, and intestines. Accumulation of the visceral WAT is highly associated with insulin resistance and diabetes. On the other hand, amount of the active BAT is negatively associated with adiposity and the likelihood of developing non-alcoholic fatty liver disease [8,9]. There are two distinct cell populations with thermogenic potential. The first population, brown adipocytes, has a common embryological origin with myocytes, and is found mainly in the intrascapular, paraspinal, supraclavicular, suprarenal, pericardial, and para-aortic regions in human. Similar to muscle, it has a high concentration of mitochondria, which gives it a characteristic brown color. The second population, known as “beige” or “brite” (“brown in white”) adipocytes, is derived from a subpopulation of white adipocytes interspersed within the WAT [10]. In human, BAT is present at birth and regresses with age, although it remains metabolically active throughout adulthood. BAT is highly innervated and vascularized, allowing it to rapidly respond to external stimulation such as cold and diet [11]. BAT also utilizes available substrates such as the intracellular triacylglycerol (TAG) and blood glucose as fuels [12].

BAT is acutely activated by cold exposure via stimulation of the sympathetic nervous system (SNS) which increases the intracellular cyclic adenosine monophosphate (cAMP) level in BAT. The high level of cAMP then increases protein kinase A (PKA)-mediated lipolysis of intracellular TAG into free fatty acids (FFA) to provide fuel for β-oxidation [6]. FFAs also β-oxidize to generate acetyl coenzyme A (acetyl-CoA), which enters the tricarboxylic acid cycle and is oxidized to generate nicotinamide adenine dinucleotide (NADH) and flavin adenine dinucleotide (FADH_2_). The NADH and FADH_2_ are then used by the electron transport system to produce a proton gradient. BAT uniquely expresses the uncoupling protein, Ucp1, which dissipates the proton gradient across the inner mitochondrial membrane, resulting in inefficiency during the formation of ATP in oxidative phosphorylation (OXPHOS). When Ucp1 is induced in the BAT, such as during cold stress or thyroid hormone (TH) stimulation, mitochondrial respiration is stimulated to the maximum amount in order to compensate for the gradient loss, generating heat in the process [6]. During sustained cold exposure, there also is adipose tissue remodeling to increase the thermogenic potential of both BAT and WAT. Chronic cold exposure causes metabolic changes in the BAT to maximize β-oxidation of fatty acids, electron transport activity, and Ucp1 expression to generate heat [6,13,14]. SNS stimulation in response to cold also induces competent white adipocytes to express Ucp1 and various BAT-associated genes such as Cidea and Cox7a1 in a process known as browning/beiging. These beige adipocytes increase their mitochondria levels and, in conjunction with the induction of Ucp1 expression, acquire the thermogenic capability [10]. The recruitment of beige adipocytes contributes substantially to heat generation during chronic cold stress [7,15]. Since they possess the capacity of fat burning, they are also considered therapeutic targets for treating metabolic dysfunction [16]. BAT activation by cold acclimation improves insulin sensitivity in patients with type 2 diabetes [17]. Since cold acclimation is able to recruit the BAT in obese patients, BAT activation may also improve obesity-associated insulin resistance and hyperglycemia [18]. The activity of adult brown and beige fat decreases with aging [19,20,21] and may contribute to the progression of chronic metabolic diseases. Taken together, these findings suggest that activating and recruiting brown and beige fat would be beneficial to improving overall metabolic health.

**Figure 1:**
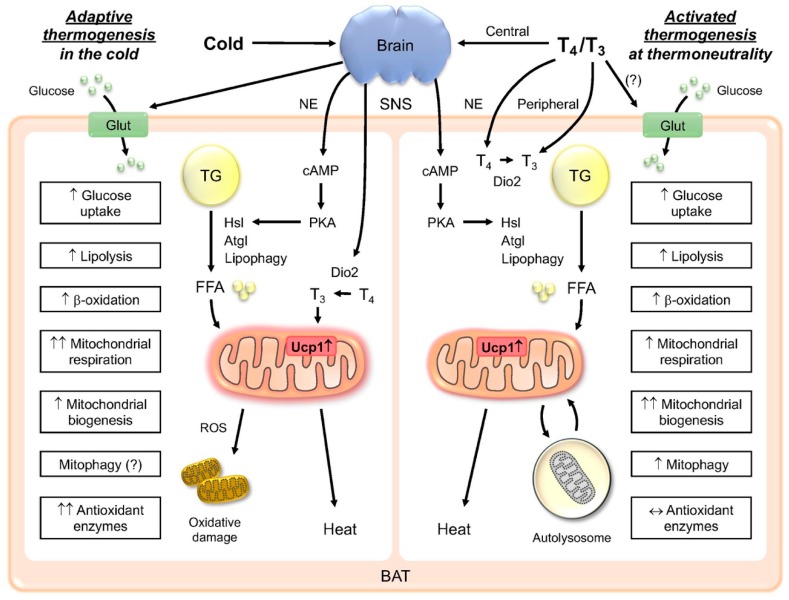
Schematic diagram of thermogenesis in the cold (adaptive) and at thermoneutrality (activated). In the adaptive thermogenesis, cold exposure stimulates the sympathetic nervous system (SNS) to release norepinephrine (NE) and induce glucose uptake and lipolysis in the brown adipose tissue (BAT). Cold exposure also increases the 5-deiodinase type 2 (Dio2) expression and intracellular conversion from T_4_ to T_3_ to facilitate the uncoupling protein 1 (Ucp1) induction and mitochondrial respiration. In the activated thermogenesis, THs (T_3_ and T_4_) induce thermogenic response centrally via the sympathetic nervous system (SNS) and peripherally via direct actions on the BAT. Similar to the adaptive thermogenesis, TH increases glucose uptake and lipolysis to provide fuel for β-oxidation. TH also directly induces Ucp1 expression in the BAT. In contrast to the adaptive thermogenesis, TH increases mitochondrial turnover by inducing both mitochondrial biogenesis and mitophagy. This leads to efficient clearance of damaged mitochondria and prevents accumulation of reactive oxidative species (ROS). Antioxidant enzymes are not upregulated in the activated thermogenesis. Glut, glucose transporter; cAMP, cyclic adenosine monophosphate; PKA, protein kinase A; TG, triacylglycerol; FFA, free fatty acids; Atgl, adipose triglyceride lipase; Hsl, hormone-sensitive lipase; ↑, upregulated; ↑↑ strongly upregulated; ↔ no change.

Interestingly, thyroid hormone (TH) and its analogs can promote tissue remodeling in both BAT and WAT by stimulating both BAT activity and browning at thermoneutral conditions. We have previously named this process “activated” thermogenesis to distinguish it from cold-induced adaptive thermogenesis [22] (Figure 1). We found that although both cold exposure and TH are able to stimulate thermogenesis in the BAT, there are inherent differences in how the metabolic processes are regulated and sustained. First, adaptive thermogenesis occurs during cold exposure, whereas activated thermogenesis induced by T_3_ occurs at thermoneutral conditions. Second, shivering occurs in conjunction with adaptive thermogenesis, whereas there is no shivering in the activated thermogenesis. Third, during cold exposure, sympathetic stimulation of the BAT plays a predominant role in BAT activation, and leads to the induction of deiodinase 2 (Dio2), the enzyme that converts T_4_ to T_3_ in the BAT during cold exposure and increases intracellular T_3_. Lopez et al. [23] showed that T_3_ acts centrally to regulate the BAT, and we also showed that local T_3_ has effects on BAT activation, so it appears that both central and peripheral stimulation are involved in the activated thermogenesis. Fourth, there is an increase in intracellular T_3_ due to the induction of Dio2 for both types of thermogenesis; however, in the adaptive thermogenesis, the induction of serum T_3_ concentration can be variable, and may depend upon the temperature and length of cold exposure. On the other hand, in the activated thermogenesis, there is a significant increase in the serum TH concentration, which also has effects on other tissues besides the BAT. In this review, we would like to describe in further detail the key similarities and differences between the TH-induced activated thermogenesis and the conventional cold-induced adaptive thermogenesis.

### 1.2. TH induction of Thermogenesis in BAT

The two forms of TH, thyroxine (T_4_) and its active metabolite 3,5,3′-triiodothyronine (T_3_), regulate obligatory and adaptive thermogenesis by directly increasing metabolic rate in specific tissues [1,24,25]. Normal thyroid status is essential for the adaptive thermogenesis in response to the cold, as hypothyroid patients are prone to hypothermia during prolonged cold exposure [26]. BAT expresses 5-deiodinase type 2 (Dio2) which catalyzes the intracellular conversion of T_4_ to T_3_ [1]. During cold exposure, there is noradrenergic induction of Dio2, which leads to an increase in intracellular T_3_ stimulation of Ucp1 expression, increased mitochondrial respiration with less efficient ATP generation, and heat generation. BAT activation by cold exposure can increase systemic T_3_ levels [27,28], suggesting that induction of Dio2 in the BAT can have some T_3_-mediated effects systemically. Cold exposure also strongly induces Dio2 expression and activity in the WAT, although its contribution to the systemic T_3_ level is not known. This induction of Dio2 by the cold in the BAT and WAT highlights the importance of T_4_ to T_3_ conversion on TH actions within adipocytes during the adaptive thermogenesis. In particular, T_3_ mobilizes triacylglycerides (TAGs) stored in the WAT to generate free fatty acids that serve as a fuel for thermogenesis. Moreover, TH is able to induce Ucp1 expression selectively in the BAT and WAT, which enables sustained induction of thermogenesis by mitochondrial uncoupling. Mice lacking Dio2 cannot maintain their body temperature during cold stress [29,30], further demonstrating that maintenance of intracellular T_3_ levels in the BAT is necessary for the adaptive thermogenesis.

Thyroid hormone receptors (TRs) belong to the nuclear receptor superfamily and are ligand-inducible transcription factors. There are two major TR isoforms, thyroid hormone receptors a and b (TRα and TRβ). Ligand-bound TRs bind to TH response elements (TREs) located in the promoter region of target genes to induce their expression. The actions of TRα and TRβ are redundant for many genes, and both TRα and TRβ are expressed in the WAT and BAT. TH regulates transcription of many thermogenic genes through its cognate receptors. However, it appears that TRα may play a more predominant role in the obligatory thermogenesis and sympathetic response, whereas TRβ may play a more significant role in stimulating Ucp1 expression in the BAT [31,32,33,34]. TRα knockout (KO), but not TRβ KO mice are hypothermic [31,35]. Administration of a TRβ-selective agonist, sobetirome (GC-1), failed to increase the cAMP level in norepinephrine (NE)-stimulated brown adipocytes from hypothyroid mice, despite normal induction of Ucp1, indicating that adrenergic response in the BAT is mostly TRα-dependent [33].

### 1.3. Central and Peripheral Regulation of Thermogenesis

During cold stress, there is strong evidence that SNS stimulates thermogenesis in the BAT [6]. However, since Dio2 expression in the BAT is necessary for thermogenesis, the local intracellular T_3_ concentration is also an important regulator of thermogenesis [29,30]. At thermoneutrality, TH also directly acts on the brain to increase the SNS activity to stimulate thermogenesis. Intracerebroventricular (ICV) administration of T_3_ decreases activity of the AMP-activated protein kinase (AMPK) and activates the lipogenic pathway in the ventromedial nucleus of the hypothalamus. This increases the sympathetic output to the BAT, as demonstrated by the increased intracellular cAMP level and thermogenic gene expression in the BAT [23]. These effects are accompanied by weight loss and may also be dependent on TH-mediated effects on autophagy in the hypothalamus. ICV administration of T_3_ increases body temperature, metabolic activity in a Ucp1-dependent manner [36]. To a lesser extent than subcutaneous administration, ICV administration of TH also induces WAT browning [37]. Taken together, these findings suggest the central action of TH plays a critical role in activated thermogenesis. However, we recently showed that T_3_ directly induces Ucp1 expression and mitochondrial activity in a BAT cell line and primary brown adipocytes suggesting that TH can also have direct effects on the BAT [22]. Additionally, it appears that autophagy (particularly, mitophagy) is critical for the TH-induced thermogenesis in the BAT as the BAT-specific autophagy-related protein 5 (Atg5) knockdown in mice abrogates the activated thermogenesis [22]. Interestingly, Mohácsik et al. used a reporter mouse that detects TH transcriptional activity, and find that TH activation of the BAT is dependent upon noradrenergic stimulation during cold exposure, but is independent of noradrenergic signaling at room temperature [38]. Therefore, both central and peripheral effects of TH are responsible for coordinated activation of thermogenesis in the BAT.

A recent study suggests that some of the thermogenic actions of TH may be Ucp1-independent. Daily subcutaneous injections of high doses of T_4_ for 3 days increases body temperature and metabolic activity in Ucp1 KO mice at thermoneutrality (23 °C) [39]. However, since BAT denervation was not performed in these studies, it is not possible to determine whether this effect is peripheral and/or central. Nonetheless, these findings suggest that there may be additional, perhaps, compensatory thermogenic effects in the BAT and/or in other tissues when Ucp1 is absent. In fact, besides heat generation, TH may also be responsible for heat conservation. Mice with a mutant TRα are unable to induce vasoconstriction of the tail, an important mechanism to prevent heat loss [40]. Together with the impairment to stimulate thermogenesis in the BAT, a further increase in heat loss could aggravate cold intolerance at the hypothyroid state. Therefore, TH can have thermogenic effects on multiple organs and tissues. Nevertheless, the effects of chronic T_4_ on metabolic rate, food intake, and thermogenic response are reduced in Ucp1 KO mice, strongly suggesting that metabolic reprogramming of the BAT occurs in these mice. In this connection, TH may facilitate Ucp1-independent thermogenesis by activating futile substrate cycles during glycolysis/gluconeogenesis, lipolysis/lipogenesis, and glycerol-3-phosphate shuttling in brown and beige fat [41,42]. A futile cycle of the arginine/creatine metabolism may also contribute to thermogenesis in the brown and beige adipocytes [43,44]. Furthermore, long-term thermogenic response, especially lipid metabolism in adipose tissues, may not require Ucp1. Grimpo et al. found that Ucp1 KO mice are able to mobilize the intracellular triglyceride (TG) in the BAT during cold adaptation, with the amount of lipid loss unrelated to uncoupled respiration [45]. Keipert el al. also show that Ucp1 KO mice have similar food intake and energy expenditure as wild-type mice when there is a gradual decrease in ambient temperature from 30 °C to 5 °C during a 5-week period [46]. Ucp1 KO mice even have a higher body temperature than wild-type mice at 5 °C, most likely due to a compensatory increase in metabolic activity in the WAT, as evidenced by a morphological browning phenotype, as well as by increased expression of browning markers such as Dio2, Pparα, and Cidea in the WAT. Taken together, these studies strongly suggest that the Ucp1-independent thermogenic response contributes to the remodeling of adipose tissue to a state of thermogenesis by its effects on lipid metabolism and mitochondrial activity.

## 2. Metabolic Actions of TH in BAT

### 2.1. TH Increases Glucose Uptake

Glucose uptake in the BAT is markedly stimulated by cold exposure [47,48] and activation of the SNS [49,50] via upregulation and/or translocation of glucose transporters [47,51,52]. In mice, TH directly increases glucose uptake in the BAT, as hyperthyroid mice increase ^18^F-fluorodeoxyglucose (^18^F-FDG) uptake in the BAT, and hypothyroid mice demonstrate the opposite effect [53]. Both the β3-adrenergic receptor agonist, BRL37344, and T_3_ also increase BAT uptake of ^18^F-FDG in control and obese mice [54]. In human, a positron emission tomography (PET) scan study of hyperthyroid Scandinavian patients shows that they have increases in glucose uptake (GU) and perfusion in BAT, WAT, and skeletal muscle [55]. On the other hand, a study of Chinese hyperthyroid patients shows that ^18^F-FDG uptake increases in skeletal muscle, but not BAT [56]. The reason(s) for the discrepancies between the two studies is not known, but could be due to differences in ethnicities of the patients and/or sensitivity of the PET scanning methods used in the studies. Finally, Skarulis et al. observed that TH activated the BAT and increased glucose disposal in a patient with extreme insulin resistance [57]. β3-adrenoceptor agonists may stimulate glucose uptake in the BAT by increasing Glut1 transcription in a cAMP-dependent manner and Glut1 translocation to the plasma membrane by mammalian target of rapamycin (mTOR) complex 2 in primary mouse adipocytes [58]. BAT expresses both glucose transporter 1 (Glut1) and the insulin-dependent Glut4. Although the expression of Glut4 is increased by TH in skeletal muscle cells [59], it is currently not known whether TH stimulates expression of glucose transporters and/or their translocation in the BAT.

It appears that adequate intracellular T_3_ concentration in the BAT is necessary for cold-induced thermogenesis in mice [29,38]. This is also evident in the clinical setting, as severely hypothyroid patients can present with hypothermia and are at increased risk of myxedema coma. Of note, ^18^F-FDG uptake in the BAT increases during cold exposure when thyroid carcinoma patients are given levothyroxine in order to render them sub-clinically hyperthyroid in order to suppress thyroid stimulating hormone (TSH) production post-surgery [60]. These findings show that exogenous levothyroxine can increase glucose uptake in the BAT during cold exposure [60], and higher levels of thyroid hormones are associated with higher amounts of cold-induced BAT activity.

### 2.2. TH Increases Fatty Acid β-Oxidation

Epinephrine or TH stimulate lipolysis of the WAT during the adaptive and the activated thermogenesis, respectively. These hormones induce expression of the adipose triglyceride lipase (Atgl) and the hormone-sensitive lipase (Hsl), the key enzymes involved in intracellular degradation of triacylglycerols. This lipolysis generates free fatty acids that can be utilized by the BAT as a fuel for thermogenesis. During the adaptive thermogenesis, induction of Ucp1 uncouples respiration from ATP production. Cold stress also increases expression of the genes involved in β-oxidation of fatty acids to generate maximum thermogenesis [61]. Significantly, pharmacological inhibition of intracellular TG lipolysis suppresses cold-induced BAT metabolism and reciprocally increases shivering in humans [62]. During the activated thermogenesis, TH plays an important role in lipid metabolism by regulating expression of the genes involved in lipid mobilization and fatty acid oxidation, including the master regulator of lipid metabolism, peroxisome proliferator-activated receptor alpha (PPARα) [25,63]. Hyperthyroid patients show increased energy expenditure and use more lipids as energy substrates [55]. Hypothyroid mice show significantly lower ^18^F-FDG and ^14^C-acetate uptake in the BAT compared to hyperthyroid mice, demonstrating that TH is indispensable for glucose utilization and lipogenesis/β-oxidation of fatty acids for thermogenesis [53,62]. Moreover, fat-specific Dio2 KO mice show a substantially higher respiratory quotient, indicating a lower contribution of fatty acid oxidation to energy expenditure [64]. Previously, we established a hyperthyroid mouse model by administering intraperitoneal T_3_ for consecutive 10 days to investigate the chronic effects of T_3_ on the BAT [22]. We found that T_3_ induces expression of the genes responsible for fatty acid oxidation (Cpt1b, Acsl1) in the BAT both in vivo and in a cell culture [22]. T_3_ also increases the levels of short- and long-chain acylcarnitines in the BAT and primary brown adipocytes. Taken together, these findings demonstrate that T_3_ acts in a direct and cell-autonomous manner to increase fatty acid β-oxidation in the BAT. Finally, TH also stimulates phosphorylation of the Atgl in the BAT, which leads to increased hydrolysis of TG and generation of fatty acids that can undergo β-oxidation within the mitochondria [22].

### 2.3. TH Increases Lipogenesis

Maintaining a healthy pool of intracellular lipids is important in order to support a high metabolic rate in the BAT. Adrenergic stimulation increases fatty acid β-oxidation and leads to rapid depletion of TG stored in the BAT [29,65]. Although cold stress increases lipolysis in the BAT and WAT, it also increases de novo lipogenesis and fatty acid re-esterification. Cold stress increases uptake of acetates into the BAT, which then is converted to acetyl-CoA to undergo either direct oxidation via the citric acid cycle or fatty acid synthesis [66]. In this regard, lipogenesis is an important anabolic process employed by the BAT to produce free fatty acids that can be used subsequently as a fuel for β-oxidation. In order to prevent detrimental induction of a hypermetabolic state at room temperature, exogenous fatty acids are preferentially packaged into diacylglycerol (DAG) or TG instead of being oxidized immediately [67,68]. Both glycerol-3-phosphate acyltransferase 4 (Gpat4) and diacylglycerol O-acyltransferase 1 (Dgat1) regulate esterification of exogenous fatty acids, while Dgat2 is responsible for esterification of the endogenous free fatty acids generated by de novo adipogenesis in the BAT [67,68]. Gpat4 homozygous knockout mice are hypermetabolic when fed a high-fat diet and oxidize 40% more exogenous fatty acids in their BAT [67]. A similar hypermetabolic phenotype is also observed in Dgat1 KO mice [69]. Dgat2 KO mice are lipopenic and die shortly after birth, apparently due to loss of substrate for energy metabolism [70]. Mice fed MEDICA-16, an inhibitor of lipogenesis, demonstrate lower core and intrascapular BAT temperature after cold exposure, further indicating that BAT lipogenesis is essential for adaptive thermogenesis [29].

TH increases lipogenesis in rats [71], as it directly stimulates transcription of lipogenic enzymes [72,73,74]. Of note, Yeh et al. show that T_3_ rescues lipogenesis as well as expression of Acc and Fas messenger RNAs (mRNAs) in the denervated BAT in hypothyroid rats [75]. In fact, T_3_ has been routinely added in the differentiation cocktail for brown adipocytes due to its direct and supportive roles in adipogenesis. TH promotes adipogenesis by regulating expression of the key adipogenic transcription factors, CCAAT/enhancer-binding protein alpha (C/EBPα) and PPARγ [76,77,78]. In this connection, 3T3-L1 cell line variants expressing mutant TRs exhibited impaired adipogenesis and reduced expression of C/EBPα and PPARγ [76]. Moreover, there was more inhibition of adipogenesis in the mutant TRα1 cell line compared to the TRβ1 mutant one, suggesting that TRα1 may play a more important role in TH-mediated adipogenesis. Consistent with this finding, a transgenic mouse model that overexpressed a mutant TRα1 also showed impaired adipogenesis and reduced transcriptional activity of PPARγ [77]. Further supporting the importance of TH in BAT differentiation, Dio2 KO embryos had a lower expression of BAT markers, Fabp4/aP2, Cidea, and Acsl5, consistent with decreased BAT adipogenesis [79]. These defects persisted even in adulthood, as these KO mice exhibited impaired adaptive thermogenesis as adults [30]. Isolated brown adipocytes from the KO mice show reduced lipolysis, Ucp1 mRNA level, oxygen consumption, as well as impaired cAMP generation in response to NE, CL316,243 (β3-adrenergic receptor-selective agonist), or forskolin [30]. These mice also have impaired lipogenesis, as they exhibit reduced induction of Acc mRNA and activity, as well as reduced lipid droplets in the BAT compared to the control mice when subjected to cold stress [29]. Consistent with these findings, an early in vitro study suggested that Dio2 is responsible for NE-stimulated lipogenesis in brown adipocytes [80]. Taken together, these data strongly suggest that T_3_-mediated lipogenesis is important for both the adaptive and activated thermogenesis.

## 3. TH effects on Mitochondria in BAT

### 3.1. TH Increases Mitochondrial Biogenesis

BAT possesses large amounts of mitochondria in order to support the high level of β-oxidation and OXPHOS induced by the SNS. Although the acute thermogenic response does not depend on mitochondrial biogenesis, increases in mitochondria content in a cell generate a higher thermogenic capacity [81]. In this connection, cold adaptation at 10 °C for 4 weeks causes a 145% increase in BAT weight in albino mice and a 185% increase in hairless mice [82]. Moreover, 7–28 days exposure to the cold increases biogenesis of thermogenic mitochondria in Djungarian hamsters [81]. The Cox subunit mRNA level and Cox activity are also increased and suggest that there is an overall increase in mitochondrial biogenesis [81]. Cold exposure or β3-adrenoreceptor stimulation induces expression of PPARγ coactivator 1-α (Pgc1α), which activates a number of transcription factors leading to increased Ucp1 and mitochondrial gene expression [83,84]. Ectopic expression of Pgc1α in adipocytes also increases the Ucp1 and mitochondrial gene expression [84]. Pgc1α KO mice were unable to maintain body temperature upon cold exposure, likely due to an impaired mitochondrial program for fatty acid β-oxidation and electron transport [85,86], further indicating the importance of Pgc1α and mitochondrial biogenesis in the adaptive thermogenesis.

TH plays a critical role in thermogenic response to the cold. TH replacement in hypothyroid patients significantly increases energy expenditure at room temperature and even more during mild cold exposure [87]. However, a study with thyroid cancer patients undergoing thyroidectomy showed that TH replacement did not consistently increase energy expenditure [88], possibly due to suppression of catecholamine synthesis and adrenergic response in the hyperthyroid state [88,89]. T_3_ and NE synergistically induce Ucp1 induction, as T_3_ is able to markedly amplify NE-stimulated Ucp1 mRNA induction in rat primary brown adipocytes [90]. Besides Ucp1 induction, TH also regulates mitochondrial protein expression and activity in the cold. Hypothyroid mice have impaired cytochrome c oxidase activity in their BAT upon cold exposure, indicating that cold alone is not sufficient to increase mitochondrial respiration [91]. On the other hand, the activity of cytochrome c oxidase in the BAT is increased by hyperthyroidism and enhanced further upon cold exposure [91]. Therefore, TH and cold exposure may have synergistic effects on mitochondrial biogenesis and activity. TH has been reported to directly induce mitochondrial biogenesis via induction of Pgc1α, Nrf1, and mitochondrial genome expression [92,93,94]. Pgc1α is also a transcriptional coactivator of TRs and is regulated by a feed forward loop that further amplifies T_3_‘s effects on gene expression [84]. In Dio2 KO mice, Pgc1α and Ucp1 expression is reduced in the BAT and mitochondrial biogenesis is impaired, which leads to a permanent defect in the adaptive thermogenesis. Insufficient intracellular T_3_ generation and reduced Pgc1α expression likely decrease mitochondrial biogenesis [79]. T_3_ injection to mice for 10 days increases mRNA and protein expression of Pgc1α as well as mitochondrial DNA and Ucp1, CoxIV, Tom20, and Vdac protein levels, suggesting that T_3_ directly regulates mitochondrial biogenesis in the BAT via Pgc1α induction [22].

### 3.2. TH Increases Mitophagy

Since BAT has a high mitochondrial content, it is not only metabolically active, but also prone to oxidative damage. Increases in the level of reduced glutathione, as well as activities of superoxide dismutase, catalase, glutathione peroxidase, and glutathione reductase suggest there is an elevated level of reactive oxidative species (ROS) in rat BAT during cold acclimation [95,96]. This increase in ROS can lead to oxidized protein and mitochondrial damage resulting in decreased mitochondrial function. In response, BAT utilizes autophagy to remove damaged organelles such as mitochondria (mitophagy). Mice with an adipose-specific impairment of autophagy have more mitochondria in their adipose tissue [97] suggesting that autophagy is essential for mitochondrial clearance in the BAT. At present, it is still controversial how autophagy in the BAT is regulated during the adaptive thermogenesis, since both autophagy inhibition and induction have been observed upon cold exposure [98,99,100]. Nevertheless, mitophagy in the BAT is important to maintain mitochondrial quality control during thermogenesis [22,99]. T_3_ directly stimulates mitophagy in the BAT in order to prevent oxidative damage in the cells. BAT-specific knockdown of Atg5 to inhibit autophagy blocks the increase in body temperature by T_3_, suggesting that autophagy in the BAT is essential for T_3_-mediated thermogenesis [22]. In summary, TH regulates coordinated mitochondrial turnover by concomitantly stimulating mitochondrial biogenesis and mitophagy. This coordinated turnover is highly efficient and sufficient to prevent ROS accumulation and protein oxidation in the BAT, since there is no concurrent induction of antioxidant enzymes (e.g., superoxide dismutase) after T_3_ treatment [22]. Currently, there are no reports on the combined effects of TH and cold on autophagy and anti-oxidant enzyme induction in the euthyroid state. It is possible that TH may ameliorate some of the cold-induced oxidative damage by promoting mitophagy, but more research is needed to determine the additive effects of TH and cold.

## 4. TH Induces Browning/Beiging in WAT

Prolonged cold exposure induces browning of a subpopulation of white adipocytes interspersed within the subcutaneous WAT [101]. This browning of white fat increases thermogenic capacity in order to maintain body temperature. TH stimulates browning/beiging by increasing mitochondrial biogenesis and Ucp1 expression [102]. Although central administration of T_3_ can mildly induce browning with increased mRNA expression of Prdm16 and Ucp1 in the WAT [37], TH also has a direct effect on WAT browning. A conjugate of T_3_ and glucagon, which selectively targets the WAT and liver, was able to induce browning in the WAT [103]. Mice rendered hyperthyroid showed an increased expression of BAT markers in their subcutaneous WAT after 3–4 weeks [53,104]. Moreover, subcutaneous administration of T_4_ for 3 weeks induced Ucp1, Pgc1α, Cidea, Prdm16 mRNA expression in the gonadal and subcutaneous WAT in rats [37]. T_3_ treatment also directly increased mitochondrial gene expression in human multipotent adipose-derived stem cells [105] and induced Ucp1 expression in a TR-dependent manner [105].

The specific role(s) of thyroid hormone receptor isoforms, TRα and TRβ, in the regulation of the BAT activity is not fully understood. The mice that lacked TRα had impaired thermogenesis during cold exposure [106] suggesting TRα was essential for temperature regulation. On the other hand, GC-1, a TRβ agonist, caused transformation of the subcutaneous WAT containing multilocular lipid droplets into brown-like adipose tissue [107]. GC-1 induced classical BAT markers Ucp1, Pgc1α, Elovl3, Cidea, Dio2, Cox5a, and CCAAT/enhancer-binding protein beta (Cebpb) [107] in the WAT. Surprisingly, GC-1 decreased Ucp1 expression and BAT glucose uptake, suggesting that browning of the WAT rather than increased BAT activity was responsible for the activated thermogenesis. However, these findings stand in contrast to other studies that showed TH increased BAT activity [22,53,54,55,57]. The discrepancy between GC-1 and TH effects on the BAT suggests that there may be potential differences in BAT activation and browning between TH and some of its analogs. TH also induced TRβ-mediated browning in the inguinal WAT, although the induction of browning did not increase glucose or triglyceride-rich protein uptake at thermoneutrality [104]. These data suggesting TRβ-selective GC-1-activated browning of the WAT and thermogenesis are difficult to reconcile with the TRα KO data showing that lack of functional TRα impaired thermogenesis during cold exposure. It is not clear whether one isoform may play a more important role in central regulation and the other in peripheral regulation of the BAT. Additionally, there may be differential roles for TR isoforms in BAT activation and WAT browning. More studies need to be performed in order to understand the mechanism(s) of browning by TH, determine the features that distinguish browning from BAT activation by TH.

## 5. Role of TH Metabolites in Thermogenesis

Although most of the studies on TH-activated thermogenesis focused on the effects of T_4_ and T_3_, the metabolites of TH can also regulate thermogenesis. 3,5-diiodo-l-thyronine (T_2_), a TH derivative, is able to activate BAT thermogenesis in hypothyroid rats [108]. Administration of T_2_ increases sympathetic innervation and vascularization of tissue, and directly increases the BAT oxidative capacity [108]. T_2_ also induces mitochondrial biogenesis by increasing the protein level of Pgc1α [108] suggesting that some of the thermogenic effects of TH may be mediated by T_2_. On the other hand, another metabolite, 3-iodothyronamine (3-T1AM), has been found to inhibit thermogenesis [109]. It induces a severe reduction in body temperature when administered to mice, likely due to tail vasodilation and increased heat loss [109]. Since the effects of TH metabolites on thermogenesis may be different from TH, more studies are needed to better understand the relevance of TH metabolites in regulating thermogenesis.

## 6. Summary

Although the classical pathway for the adaptive thermogenesis in the BAT has been well studied, the role of TH in thermoregulation is only partially understood. Sympathetic activation appears to be the main driver for thermogenic activation; however, the induction of Dio2 in the BAT and WAT during cold exposure strongly implicates an indispensable role of intracellular T_3_ in adipose tissues during thermogenesis. While some studies have suggested that the central action of TH is sufficient for thermogenic activation of the BAT, peripheral TH may also be involved in cold adaptation by its effects on metabolic remodeling of the BAT and WAT. TH can also induce thermogenesis at thermoneutrality, which we term “activated” thermogenesis (Figure 1). It involves TH-mediated SNS stimulation of the BAT as well as peripheral actions by TH. TH stimulation of the BAT promotes utilization of glucose and fatty acids as fuels, with the latter playing the predominant role during chronic stimulation. TH also promotes autophagy, which facilitates mitochondrial turnover during the activated thermogenesis. In summary, TH and various TH analogs can increase the thermogenic potential of the BAT and WAT by inducing fatty acid β-oxidation, lipogenesis, mitochondrial biogenesis, and autophagy. Careful titration of TH and targeted delivery (e.g., using TH analogs preferentially taken up by the BAT) may reduce the adverse effects of TH on other tissues, such as heart and bone. Given the current obesity epidemic, stimulation of the BAT function and browning of the WAT by TH suggest that TH or its analogs could be promising therapeutic agents to increase energy expenditure and counteract weight gain.

## Abbreviations

Atg5	Autophagy-related protein 5
BAT	Brown adipose tissue
cAMP	Cyclic adenosine monophosphate
DAG	Diacylglycerol
Dgat1	Diacylglycerol O-acyltransferase 1
Dio2	5-Deiodinase type 2
FADH_2_	Flavin adenine dinucleotide
18F-FDG	18F-Fluorodeoxyglucose
FFA	Free fatty acids
Gpat4	Gycerol-3-phosphate acyltransferase 4
Glut	Glucose transporter
KO	Knockout
mTOR	Mammalian target of rapamycin
NADH	Nicotinamide adenine dinucleotide (NADH)
OXPHOS	Oxidative phosphorylation
PET	Positron emission tomography
PKA	Protein kinase A
TH	Thyroid hormone
TR	Thyroid hormone receptor
TG	Triacylglycerol
Ucp1	Uncoupling protein 1
SNS	Sympathetic nervous system
WAT	White adipose tissue
